# pubmed2ensembl: A Resource for Mining the Biological Literature on Genes

**DOI:** 10.1371/journal.pone.0024716

**Published:** 2011-09-29

**Authors:** Joachim Baran, Martin Gerner, Maximilian Haeussler, Goran Nenadic, Casey M. Bergman

**Affiliations:** 1 Faculty of Life Sciences, University of Manchester, Manchester, United Kingdom; 2 School of Computer Science, University of Manchester, Manchester, United Kingdom; University of Leuven, Belgium

## Abstract

**Background:**

The last two decades have witnessed a dramatic acceleration in the production of genomic sequence information and publication of biomedical articles. Despite the fact that genome sequence data and publications are two of the most heavily relied-upon sources of information for many biologists, very little effort has been made to systematically integrate data from genomic sequences directly with the biological literature. For a limited number of model organisms dedicated teams manually curate publications about genes; however for species with no such dedicated staff many thousands of articles are never mapped to genes or genomic regions.

**Methodology/Principal Findings:**

To overcome the lack of integration between genomic data and biological literature, we have developed pubmed2ensembl (http://www.pubmed2ensembl.org), an extension to the BioMart system that links over 2,000,000 articles in PubMed to nearly 150,000 genes in Ensembl from 50 species. We use several sources of curated (e.g., Entrez Gene) and automatically generated (e.g., gene names extracted through text-mining on MEDLINE records) sources of gene-publication links, allowing users to filter and combine different data sources to suit their individual needs for information extraction and biological discovery. In addition to extending the Ensembl BioMart database to include published information on genes, we also implemented a scripting language for automated BioMart construction and a novel BioMart interface that allows text-based queries to be performed against PubMed and PubMed Central documents in conjunction with constraints on genomic features. Finally, we illustrate the potential of pubmed2ensembl through typical use cases that involve integrated queries across the biomedical literature and genomic data.

**Conclusion/Significance:**

By allowing biologists to find the relevant literature on specific genomic regions or sets of functionally related genes more easily, pubmed2ensembl offers a much-needed genome informatics inspired solution to accessing the ever-increasing biomedical literature.

## Introduction

Advances in DNA sequencing technology over the last 40 years have drastically increased the rate of genomic sequence data production (http://www.ncbi.nlm.nih.gov/genbank/genbankstats.html), which has in turn directly accelerated the rate of biological discovery and publication (http://www.nlm.nih.gov/bsd/index_stats_comp.html). Genomic sequences and their supporting computational annotations are now well served by generic genome portals, such as the Ensembl [1] or UCSC [2] genome browsers, or by specific model organism databases, such as FlyBase [3] or the *Saccharomyces* Genome Database [4]. Substantial effort has been invested in integrating these genomic database resources with other gene-based microarray or protein resources, such as ArrayExpress [5] or UniProt [6], which are now highly interoperable with genome and model organism databases. In contrast, very little effort has been made to systematically integrate genomic data directly with the biological literature [7], despite the fact that these are the two most heavily relied-upon sources of information for many biologists.

The primary portal for access to biomedical literature is PubMed [8], the US National Library of Medicine's (NLM) searchable interface to the MEDLINE database. Integrating papers in PubMed/MEDLINE with genomic data would be trivial if authors used standardized genome database identifiers to refer to gene names in text. Unfortunately, this is not the case and genes are referred to in the literature by many variant spellings or synonyms [9], [10]. Major advances have been made over the last few years in the field of automated gene name recognition and mapping of gene names to database identifiers [11]. However, automated text-mining systems have not yet been integrated into the MEDLINE indexing process (http://www.nlm.nih.gov/bsd/indexing/training/USE_010.htm). Thus, while some important genes are curated as part of the MeSH controlled vocabulary (see terms under MESH tree node G05.360.340.024.340), the comprehensive assignment of specific gene or genomic regions to PubMed/MEDLINE records is currently not part of the centralized warehousing of the biological literature. As a result, thousands of primary research articles in PubMed that refer to specific regions in sequenced organisms are not directly linked to any genomic data.

For a limited number of model organisms (*S. cerevisiae*, *Drosophila melanogaster*, *Caenorhabditis elegans*, *Arabidopsis thaliana*, *Mus musculus*, etc.) dedicated teams of human curators scan the biological literature for publications that contain references to species-specific gene names, and subsequently link PubMed document IDs (PMIDs) to gene records in model organism databases such as SGD, FlyBase, WormBase, TAIR and MGI [12]. Researchers typically access these curated links between genes and the literature on a gene-by-gene basis using web-interfaces specific to each model organism database, and thus these data are often not amenable to bulk or integrative data mining. NCBI's Entrez Gene project [13] integrates these externally-curated and other internally-generated data sources (such gene-PMID links obtained from GenBank records) and provides limited web and programmatic interfaces for searching papers linked to genes. However, the ever-increasing rate of publication often necessarily creates a backlog of uncurated articles, or requires curation teams to adopt triage procedures whereby only a subset of the literature is curated and linked to gene and genome resources [12]. Finally, many thousands of articles refer to genes in sequenced genomes for species with no such dedicated literature curation staff, and thus may never be mapped to genes or genomic regions by human curators.

To help overcome the lack of integration between the genomic information and the biomedical literature, we have developed pubmed2ensembl (http://www.pubmed2ensembl.org), a database and set of interfaces based on an extended version of the BioMart data mining system [14] that provides more than 2,000,000 links between genes and publications from six curated and automatically generated data sources. Using BioMart's powerful built-in web and programmatic interfaces, researchers can flexibly query, constrain and combine different literature and genomic data sources to suit their particular research needs. Furthermore, we have extended BioMart's MartView web interface to permit text search queries on PubMed or PubMed Central (PMC), where the result set of documents from the query can be applied as a filter to constrain gene-publication links in our database. By providing the much-needed ability to perform queries using both textual and genomic constraints, pubmed2ensembl should aid experimental and computational researchers alike by establishing bridges between two of the fastest growing sources of biological information.

## Materials and Methods

### Overview of the BioMart 0.7 data mining framework

In order to develop a database that links genes to publications with a powerful and user-friendly front end for constructing queries, we chose to design pubmed2ensembl on top of a customized version of the BioMart 0.7 system [14]. BioMart is an open-source data integration and analysis framework that provides tools to create and build an optimized database out of one or more relational databases. Here we briefly summarize the BioMart 0.7 build process and query interfaces to provide a foundation for the extensions to BioMart 0.7 we developed as a part of pubmed2ensembl (see Results).

A BioMart is conventionally built using MartBuilder, a GUI tool that allows users to select and link tables of one or more databases that will be rewritten into a BioMart, and also produces the necessary SQL commands that are required to create the actual BioMart. The SQL commands can then be executed using MartRunner, another GUI tool that supports running SQL commands in parallel in order to reduce the time required to generate the BioMart. The MartEditor tool is then used to describe which columns of the BioMart's SQL tables should be accessible through the various interfaces as attributes and filters. MartEditor also allows for the customization of how the results are rendered in BioMart's MartView interface. For some BioMarts, such as the Ensembl BioMart that we extend here, the underlying data is partitioned into several datasets prior to the build process (e.g. one dataset for each species within the Ensembl database) to allow subsequent query optimization.

Once created, the resulting BioMart database can then be queried through the various BioMart interfaces, including: (i) MartView: an interactive web-interface for constructing database queries and displaying query results; (ii) MartExplorer: an application tailored for accessing BioMarts; (iii) MartShell: a command line interface to query BioMarts; (iv) MartService: a RESTful interface for programmatic BioMart access; and (iv) a built-in Distributed Annotation System (DAS) server. Queries in BioMart are formulated over “attributes” (the items that are selected by the query), where “filters” can be used to constrain the query result. An attribute denotes a named column in a table of the BioMart database. Filters can be applied to attributes, limiting the results that are returned to certain values or value ranges.

By using the BioMart framework for pubed2enesmbl, it is possible to query gene-publication data sources in pubmed2ensembl *via* any of the above interfaces with no additional software development. Furthermore, a number of popular external software systems (such as Galaxy [15], Bioconductor [16], and Taverna [17]) have implemented methods to query and import data directly from BioMart instances [14], and thus data and results from pubmed2ensembl can be automatically integrated into these systems as well.

### Data sources used to create pubmed2ensembl

To provide links between the literature and genes for data exploration in pubmed2ensembl, we extended the existing Ensembl BioMart with six additional data sources that link Ensembl gene IDs to PMIDs: Entrez Gene, gene name recognition in MEDLINE abstracts and PMC full-text articles, mapping of sequences in EMBL records to Ensembl transcripts, database cross-references in EMBL records to genes in Ensembl, and gene-PMID mappings from the text2genome database. The following provides details of the import process for each of these six data sources in pubmed2ensembl.

#### Entrez Gene

We imported curated gene-PMID links from the Entrez Gene database [13], which contains published information about genes that appear as part of the International Nucleotide Sequence Database Collaboration (DDBJ, EMBL and GenBank), NCBI's Reference Sequence collection, genes that are part of certain recognized external genome databases, and contributions from external users and NLM staff. Specifically, we directly imported the contents of a snapshot of Entrez Gene data (12 April 2010) from the gene2pubmed TSV file (ftp://ftp.ncbi.nlm.nih.gov/gene/DATA/gene2pubmed.gz). Links between genes and publications are stored over three columns, denoting NCBI taxonomy ID, Entrez Gene ID and PMID respectively, which we imported directly into pubmed2ensembl.

#### Gene Name Recognition in MEDLINE and PMC

We automatically generated gene-PMID links by using a text mining system that recognizes gene name mentions in MEDLINE abstracts and full-text PMC articles and normalizes these mentions to standardized gene identifiers. Normalized gene mentions were then linked to their documents' PMIDs. To do this, we used GNAT (version 21 January 2009) [18] in conjunction with the LINNAEUS species identification system (version 1.4) [19], where the LINNAEUS component aided GNAT in cross-species gene identification. We used GNAT since it is one of the best-performing gene name normalization systems [11], [18], and currently is one of the few systems that is publicly available for large-scale text-mining. The version of GNAT that we used is capable of finding genes for 18 model organisms [18], but only ten of these are part of Ensembl 56. Gene name normalization was carried out on the MEDLINE 2010 baseline corpus of 10,240,192 abstracts and a snapshot of 186,598 full-text articles from the Open Access (OA) subset of PMC taken on 5 February 2009. GNAT normalizes to Entrez Gene IDs, and thus for MEDLINE abstracts we imported a table containing links between NCBI taxonomy IDs, Entrez Gene IDs and PMIDs. For PMC articles, PMCIDs were converted to PMIDs (see below) and a table with NCBI taxonomy IDs, Entrez Gene IDs, PMCIDs and PMIDs was imported into pubmed2ensembl.

#### Linking sequences in EMBL records to transcripts in Ensembl using BLAST

We automatically generated gene-PMID links from the EMBL nucleotide databank [20] by extracting EMBL records that contain a PMID and using the corresponding DNA sequences in a BLAST search against a database of transcripts from Ensembl genes, with subsequent filtering for promiscuous BLAST matches. Searches of nucleotide sequences from the “standard” dataset (STD) of EMBL 102 containing PMIDs were carried out on Ensembl 56 transcripts (which included both transcript and gene IDs in their fasta headers) *via* NCBI blastall (version 2.2.18) (ftp://ftp.ncbi.nlm.nih.gov/blast/) with the parameters: -p blastn -a 8 -m 8 -e 10 -b 1 -G 1 -E 1 -F “m D”. We subsequently improved the quality of the data in two filtering steps: first, we remove PMIDs that appear in 100 or more EMBL records in order to increase the specificity between gene-PMID matches; and second, we remove PMIDs whose EMBL records refer to the regular expressions ‘mir-*’, ‘mitochond*’, ‘tRNA’, ‘ribosom*’ or ‘plasmid*’ in either title- or keyword-sections of a record to reduce the false positive gene-PMID associations. The resulting set of filtered BLAST hits was used to create a table with taxonomy IDs, EMBL IDs, PMIDs, Ensembl Gene IDs that was imported into pubmed2ensembl.

#### Extraction of Ensembl database cross-references from EMBL records

We automatically generated gene-PMID links from EMBL records using annotated cross-references (XREFs) to genes for species whose gene models are produced by the Ensembl pipeline. Where both PMID and Ensembl Gene IDs were annotated, we extracted and recorded the gene-PMID links. To do this, records from the “standard” (STD) subset of EMBL-Bank Release 102 were parsed for explicit mentions of both PMIDs and Ensembl Gene IDs, and the resulting set of taxonomy ID, EMBL ID, PMID, Ensembl Gene ID links were output as a TSV file and imported into pubmed2ensembl.

#### text2genome

Finally, we directly imported data from the text2genome database [21], which generates gene-PMID links based on nucleotide sequences extracted from full-text articles that are mapped to genes by aligning extracted sequences to Ensembl transcripts. Data from text2genome were obtained from the same archive of full text articles from PMC OA of 5 February 2009 as used in the gene name recognition analysis above. Extracted sequences were aligned to Ensembl transcripts using BLAT with default parameters with subsequent filter to retain only the first best-scoring alignment (using the UCSC tools pslSort and pslUniq) (see [21] for details). PMCIDs were converted to PMIDs (see below) and a table of Ensembl Gene IDs, PMIDs, and PMCIDs was imported.

### PMCID to PMID conversion

PMCIDs can be converted to PMIDs *via* data provided by the NLM (ftp://ftp.ncbi.nlm.nih.gov/pub/pmc/PMC-ids.csv.gz). However, some publications in PMC are not indexed by MEDLINE and therefore are not available in PubMed, and for some articles the PMCID is not mapped to a PMID yet. Because of these discrepancies, 26,231 out of 2,126,080 PMCIDs from the gene name recognition on PMC are not mapped to PMIDs. All PMCIDs are mapped to PMIDs for data from text2genome. We note that even though we are providing PMCIDs for some data sources where they were part of the original data source (gene name recognition in PMC and text2genome), results presented in this paper only use PMIDs.

### pubmed2ensembl BioMart creation

While providing powerful tools for BioMart construction, the GUI-base nature of the MartBuilder/MartRunner/MartEditor trio of tools prevents reproducible offline construction of BioMart in a fully automated manner. Therefore, we created and implemented a scripting language, named MartScript, which allows for the automated and reproducible creation of a BioMart. MartScript is available under open-source licenses at https://github.com/pubmed2ensembl/MartScript. MartScript allows the interactive steps that would previously be carried out using the MartBuilder, MartRunner and MartEditor GUIs described above to be carried out automatically from the command line. In principle, to achieve this aim we could have further developed the MartShell scripting language provided with BioMart 0.7 that is used to query and update constructed BioMarts. However, we chose to implement out own scripting language to ensure control over its design and implementation. Currently MartScript is tailored to work only with Ensembl ‘core’ and ‘vega’ BioMart database tables (although it can be extended to any database in principle).

**Figure 1:**
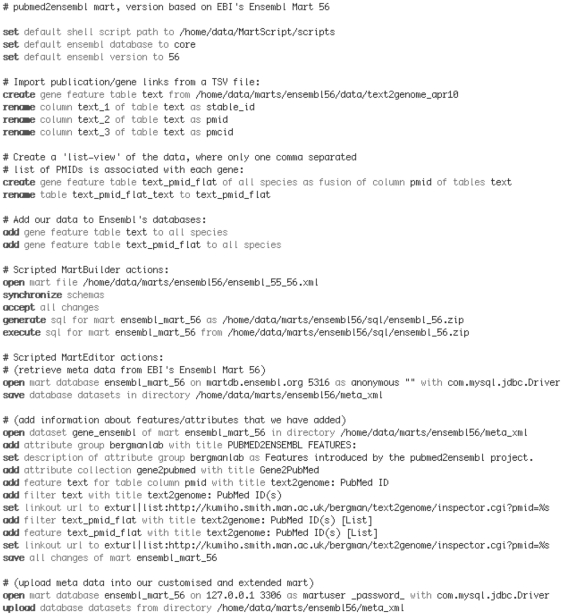
An example MartScript for automatically importing data and creating a pubmed2ensembl BioMart. MartScript commands shown describe how an external data source is imported and added to the BioMart build process, how the SQL commands for the actual BioMart creation are generated and executed, and how meta-data that describe filter and attribute names are imported and customized into pubmed2ensembl. The example script is augmented with comments that explain the semantics of the commands.

MartScript is an imperative programming language without branching (i.e. if-then-else constructs are intentionally not supported). In order to simplify the language, its syntax has been designed to resemble written English. An excerpt of the script used to create the pubmed2ensembl BioMart is shown in Figure 1, demonstrating how an external data source is imported and added to the BioMart build process, how the SQL commands for the actual BioMart creation are generated and executed, and how meta-data that describe filter and attribute names from the official Ensembl BioMart are imported and customized into pubmed2ensembl. When executed, the complete script will automatically integrate our gene-publication data sources with a core Ensembl database to create a pubmed2ensembl instance.

We applied MartScript to the ‘core’ and ‘vega’ database tables from Ensembl Release 56 together with the six additional pubmed2ensembl data sources described above. All our six data sources were imported from TSV files that have a common structure, with the first column holding the species taxonomy ID, and the second column holding either an Entrez Gene ID or Ensembl Gene ID unless otherwise specified by MartScript. MartScript statements override default locations of gene IDs to specify the identity of the remaining columns, including those for PMIDs (integers) and PMCIDs (string starting with “PMC”). Further columns hold additional information that is used by MartScript to create supplementary-data pop-ups in the MartView web interface that are reformatted and escaped for HTML accordingly. MartScript automatically maps Entrez Gene IDs to Ensembl Gene IDs via cross-references in the object_xref table that are present at the transcript level, so that the data can be successfully integrated into the Ensembl databases before the actual SQL commands for the BioMart creation are generated.

### Data analysis

In order to generate the results presented here, local pubmed2ensembl SQL tables were queried directly to extract lists of PMIDs, Ensembl Gene IDs and Ensembl Gene ID-PMID pairs. Unique lists of IDs were then generated for each source (across all species), each species (across all sources) and for the entire dataset (across all species and sources), to remove redundancy across different species and sources since some papers can refer to genes in more than one species. Intersections of ID lists were then calculated for each unique data source (across all species). Gene-publication links from human-curated BioCreAtIvE I (task 1b) and II Gene Normalization training and testing datasets [11], [23] were mapped, where possible, to Ensembl Gene ID and PMIDs and compared to gene-PMID pairs from pubmed2ensembl. Data manipulations were performed using custom GNU bash and PERL scripts.

## Results

In the following we first describe the customization we made to the search interfaces of BioMart 0.7 that we have included in pubmed2ensembl. We then provide an overview of the contents of the pubmed2ensembl database. Finally, we provide example use cases to demonstrate the features and utility of the pubmed2ensembl system.

### An extended BioMart 0.7 data mining framework

We made several modifications to the basic BioMart search interfaces in order to customize them to better serve our gene-publication data. These modifications are available under open-source licenses at https://github.com/pubmed2ensembl/biomart-plus-extras. First, we extended BioMart's MartView interface to permit text searches of the PubMed and PMC databases, which return PMID lists that can automatically be used as a filter on our data sources. Second, we introduced visualization aids that improve the readability of MartView result sets. Third, we extended BioMart's built-in DAS server to export gene-related publication information as DAS tracks to archived versions of the Ensembl Genome Browser.

#### Integration of PubMed/PubMed Central text search into MartView

**Figure 2:**
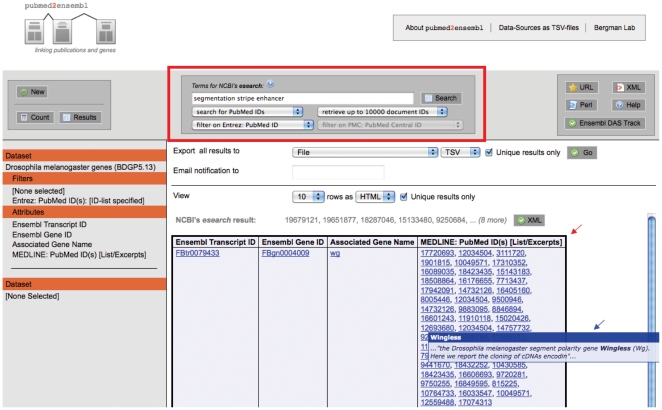
Screenshot of the extended MartView interface implementing NCBI eUtilities search and other visualization improvements. The central search box (outlined in red) provides new functionality to BioMart that allows integrated queries over text in PubMed/PubMed Central and genomic data in Ensembl. Results can also be displayed in a “list” format in a single row (red arrow), and publications linked to genes *via* gene name recognition show an excerpt of the underlying textual evidence as a pop-up window (blue arrow).

To develop a search interface that permits queries on text and genomic constraints, we extended the MartView web interface to allow users to execute a text search on NLM's PubMed and PMC databases *via* the Entrez Progamming Utilities (eUtils) (http://eutils.ncbi.nlm.nih.gov/entrez/query/static/eutils_help.html) (Figure 2). The PMID lists returned by such a search can then be used as a filter on PMIDs in pubmed2ensembl data sources, which will then restrict the results of a pubmed2ensembl BioMart query to information related to the original text query. Our design choice of using a remote search of PubMed/PMC *via* eUtils coupled with local filtering on a resulting PMID set prevents us from having to index and search PubMed or PMC locally on our server.

#### Improvements to the layout of MartView results

For queries that produce one-to-many relations, MartView presents results in a non-intuitive way since results are spread over multiple rows. This is a problem for data like that in pubmed2ensembl, since genes are often linked to multiple PMIDs. The expansion of gene-PMID relations becomes even more problematic when two or more of our PMID sources are displayed simultaneously. In order to compress the tabular output from a query to a minimum, we introduced an automatic rewriting process that is carried out by MartScript, which condenses the PMIDs linked to a gene into a comma-separated list. In this alternative representation, what would otherwise be expanded rows of PMIDs will be collapsed into fewer rows (Figure 2). Link-out URLs (e.g. from PMIDs to PubMed abstracts) are still supported in the list format. Additionally, for data sources that use gene name recognition text mining software, we have included functionality to activate a pop-up that shows the text immediately surrounding gene mentions when a user hovers over PMIDs of papers linked to genes (Figure 2).

#### Extension of the BioMart DAS server display

While in principle it is possible to use the built-in BioMart DAS server to display pubmed2ensembl data on the current Ensembl genome browser, a certain amount of time is required to gather and prepare a pubmed2ensembl BioMart build, making it difficult to continually support the latest version of the Ensembl genome database. Since BioMart 0.7 does not support the DAS ‘sources’ command, which is part of the DAS 1.53E specification [22], it is not possible to link DAS tracks of BioMart 0.7 to archived versions of the Ensembl genome browser. Therefore, we extended BioMart 0.7 to include a DAS sources command in order to provide a DAS track on the Ensembl browser archive that shows information about linked publications for each gene. In our extended MartView interface, we provide link-outs to the Ensembl genome browser archive on a per species basis, where the DAS track is automatically configured and attached to the genome browser. For each item on the DAS track, the total number of PMIDs for each of the six data sources is displayed and a HTTP-link is provided back to the pubmed2ensembl MartView interface with a filter on the gene in question to display the data for a particular gene.

### Summary of the pubmed2ensembl database contents

In total, we mapped 2,093,066 PMIDs to 148,019 Ensembl genes in 5,459,005 unique gene-PMID links from the six data sources we have included in pubmed2ensembl. Table 1 summarizes the number of PMIDs, genes, and gene-PMID links, respectively, for the six data sources of gene-PMID links individually. Summaries of the number of PMIDs, genes and gene-PMID links for individual species across individual data sources can be found in Supplementary Files S1, S2 and S3, respectively. Each paper in pubmed2ensembl is linked to a mean (median) of 2.6 (1) gene(s) while each gene is linked to a mean (median) of 36.8 (4) papers. Clearly, the mean number of papers per gene is upwardly biased by very heavily-studied genes such as human TNF-α (ENSG00000223952), which has over 49,000 linked publications. Likewise, the mean number of genes per paper is upwardly biased by papers reporting genome sequence or large-scale cDNA projects that are linked to many genes, such as PMID:12477932, which is linked to over 65,000 genes. Overall, only 11.3% of publications with a PubMed ID could be linked to genes in Ensembl (2,093,066 out of 18,502,916) and only 13.6% of genes in Ensembl were linked to publications (148,019 out of 1,090,540).

**Table 1:** Total number of PMIDs, gene IDs, and gene ID-PMID pairs for all data sources in the pubmed2ensembl database.

Source	PMIDs	Gene IDs	Gene ID-PMID pairs
Entrez	469,872	102,415	1,652,017
MEDLINE	1,867,773	36,310	3,439,750
PMC	102,405	25,870	635,885
EMBL BLAST	69,764	64,335	129,530
EMBL XREF	28,982	82,940	170,722
text2genome	9,128	11,560	24,188
Total (non-redundant)	2,093,066	148,019	5,459,005

Note that PMC and text2genome data sources only include documents in the OA subset of PMC.

The overwhelming majority of PMIDs linked to genes in pubmed2ensembl come from GNAT gene name recognition on MEDLINE abstracts, followed distantly by PMIDs imported from Entrez Gene (Table 1). All other data sources provide more than an order of magnitude fewer PMIDs than MEDLINE. However, despite providing the largest number of publications linked to genes, gene name recognition on MEDLINE does not contribute the greatest number of genes in pubmed2ensembl. Entrez Gene, EMBL XREF and EMBL BLAST all provide links to papers for a larger number of genes than gene name recognition on MEDLINE. Numbers of gene-PMID links follow the same trend as PMIDs, with the greatest number of specific gene-PMID links coming from the MEDLINE and Entrez Gene sources. We note that the number of PMIDs, genes and gene-PMID links obtained from text mining on MEDLINE is limited by the fact that only 55% of all MEDLINE records have associated abstracts (10,240,192 out of 18,502,916).

**Figure 3:**
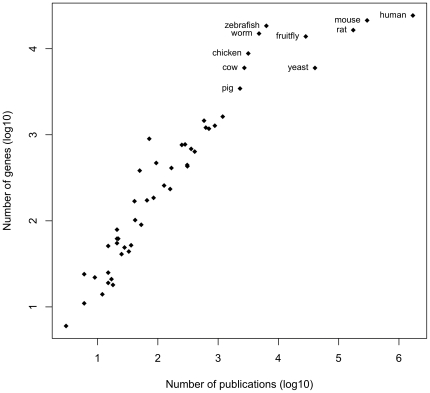
Relationship between the number of publications and genes summed across all data sources in the pubmed2ensembl database plotted by species. The ten species with largest total numbers of publications and genes linked are labeled with their common names.

Figure 3 shows the number of publications and genes summed across all data sources plotted by species. As expected, human is the species with the greatest number of publications linked to genes, followed by common model organisms (mouse, rat, yeast, fruitfly, zebrafish and worm) and important agricultural species (chicken, cow and pig) (cf. [19]). While the number of genes for the top ten organisms ranges within one order of magnitude, the number of papers for the top ten organisms spans nearly three orders of magnitude, underscoring the extreme density of published information on human, mouse and rat genes even relative to other well-studied species. As shown in Supplementary Files S1, S2, and S3, we find that sources that use sequence mapping have the broadest coverage across species (EMBL BLAST, n = 50 species; text2genome, n = 30 species), while sources that used database cross-references had intermediate coverage (Entrez Gene, n = 20 species; EMBL XREF, n = 26 species), and sources that used gene name recognition had the narrowest coverage (MEDLINE and PMC, 8 species each) (see Discussion).

To assess the consistency of gene-PMID mappings inferred using different approaches, we compared the number (Table 2) and percentage (Table 3) of overlaps between gene-PMID pairs across all data sources. It is less meaningful to assess overlaps on the PMID or gene level alone, since PMIDs or genes could be shared among data sources even though they do not support the same gene-PMID link. We find that while there are large numbers of specific gene-PMID links that are supported by more than one data sources (Table 2), gene-PMID links found in more than one data source make up only a relatively small proportion of the total gene-PMID links for any particular data source (Table 3). For example, only 22.2% of Entrez Gene links are supported by automated gene name recognition in MEDLINE, and all other data sources support less than 6% of Entrez gene-PMID links. Levels of overlap can in part be interpreted by how closely different data source use the same material to establish gene-PMID links. In general, lower levels of overlap are observed for data sources that use different materials (e.g. gene name recognition on MEDLINE uses only abstracts, while curated links from Entrez Gene may use the full text article). The highest level of consistency between sources observed is for Entrez Gene and EMBL (BLAST or XREF) where, relative to the total number of EMBL gene-PMID links, approximately half are found in Entrez Gene. This is likely a result of the regular data exchange between EMBL and GenBank coupled with the fact that Entrez Gene imports links between genes and PMIDs from GenBank records. Similarly, a relatively high proportion of the text2genome gene-PMID links are supported by gene name recognition on PMC [21], which both use the same PMC full-text articles as their underlying data source.

**Table 2:** Number of overlapping gene-PMID pairs found in pairwise combinations of data sources in the pubmed2ensembl database.

	MEDLINE	PMC	EMBL BLAST	EMBL XREF	text2genome
Entrez	365,970	16,876	61,245	94,358	4,234
MEDLINE	-	43,022	28,785	15,559	4,004
PMC	-	-	1,195	516	9,779
EMBL BLAST	-	-	-	39,203	1,434
EMBL XREF	-	-	-	-	489

**Table 3:** Degree of overlap among gene-PMID pairs in different pairwise combinations of data sources in the pubmed2ensembl database.

	Entrez	MEDLINE	PMC	EMBL BLAST	EMBL XREF	text2genome
Entrez	-	22.2%	1.0%	3.7%	5.7%	0.3%
MEDLINE	10.6%	-	1.3%	0.8%	0.5%	0.1%
PMC	2.7%	6.8%	-	0.2%	0.1%	1.5%
EMBL BLAST	47.3%	22.2%	0.9%	-	30.3%	1.1%
EMBL XREF	55.3%	9.1%	0.3%	23.0%	-	0.3%
text2genome	17.5%	16.6%	40.4%	5.9%	2.0%	-

Numbers reflect the percentage of overlapping gene-PMID pairs for a column-row combination, relative to the total number of gene-PMID pairs from the data source on the left-most column. For example, gene-PMID pairs found in both Entrez Gene and MEDLINE comprise 22.2% of the total number of pairs from the Entrez source but only 10.6% of the total number of pairs from the MEDLINE source.

To evaluate the quality of the data in pubmed2ensembl, we compared gene-PMID links from each of the six pubmed2ensembl data sources to gene-PMID links from the BioCreAtIvE I and II Gene Normalization challenges [11], [23]. The BioCreAtIvE organizers provide human-curated training and testing data sets of normalized gene mentions linked to document IDs for evaluating gene normalization software. These data are derived from a total of 1,744 MEDLINE abstracts of publications discussing human, mouse, fruitfly and yeast genes. Where possible, we converted BioCreAtIvE data sets into a standardized format including Ensembl Gene IDs and PMIDs, which resulted in reduced dataset of 1,671 abstracts containing a total of 5,326 gene-PMID links. We evaluated whether individual gene-PMID links were found in pubmed2ensembl data sources for each of these species (Supplementary Files S4, S5, S6, and S7) and estimated the precision and recall for each data source on a species-specific basis (Table 4). In general, we found that the MEDLINE, EMBL BLAST and text2genome data sources exhibited relatively high precision relative to the BioCreAtIvE datasets. Precision of the Entrez Gene, PMC and EMBL XREF data sources was high for some species but low for others. Recall for all pubmed2ensembl data sources was in general low, with the exception of Entrez Gene which achieved reasonable recall for human and mouse and very high recall for fruitfly.

**Table 4:** Precision and recall of pubmed2ensembl data sources relative to the BioCreAtIvE I and II Gene Normalization data sets.

Species	Source	PMIDs	gene-PMIDpairs	TP	FP	FN	Precision	Recall
human	Entrez	529	1,393	831	562	585	0.597	0.587
	MEDLINE	431	1,059	898	161	518	0.848	0.634
	PMC	5	45	13	1,046	1,403	0.289	0.009
	EMBL BLAST	348	517	385	132	1,031	0.745	0.272
	EMBL XREF	279	406	300	106	1,116	0.739	0.212
	text2genome	2	2	2	0	1,414	1.000	0.001
	Total	531	1,416	-	-	-	-	-
mouse	Entrez	469	7,248	983	6,265	311	0.136	0.760
	MEDLINE	317	456	428	28	866	0.939	0.331
	PMC	4	8	7	449	1,287	0.875	0.005
	EMBL BLAST	98	148	110	38	1,184	0.743	0.085
	EMBL XREF	46	57	53	4	1,241	0.930	0.041
	text2genome	1	1	1	0	1,293	1.000	0.001
	Total	481	1,294	-	-	-	-	-
fruitfly	Entrez	308	1,511	1,442	69	124	0.954	0.921
	MEDLINE	45	58	53	5	1,513	0.914	0.034
	PMC	2	6	2	56	1,564	0.333	0.001
	EMBL BLAST	67	84	74	10	1,492	0.881	0.047
	EMBL XREF	0	0	0	0	1,566	N/A	0.000
	text2genome	2	4	4	0	1,562	1.000	0.003
	Total	314	1,566	-	-	-	-	-
yeast	Entrez	116	393	300	93	750	0.763	0.286
	MEDLINE	230	393	384	9	666	0.977	0.366
	PMC	14	47	23	370	1,027	0.489	0.022
	EMBL BLAST	42	52	42	10	1,008	0.808	0.040
	EMBL XREF	0	0	0	0	1,050	N/A	0.000
	text2genome	1	2	1	1	1,049	0.500	0.001
	Total	345	1,050	-	-	-	-	-

Gene-PMID pairs found in both a pubmed2ensembl data source and a BioCreAtIvE I and II Gene Normalization data sets were considered true positives (TP). If a gene-PMID pair was present in a pubmed2ensembl data source but not in a BioCreAtIvE data set, it was considered a false positive (FP). Conversely, if a gene-PMID pair was not present in a pubmed2ensembl data source but was present in a BioCreAtIvE data set, it was considered a false negative (FN). Precision was calculated as TP/(TP+FP); Recall was calculated as TP/(TP+FN). Rows labeled with Total indicated the total number of PMIDs and gene-PMID pairs in the BioCreAtIvE evaluation data set for each species. Values with division by zero are labeled “N/A”.

### Example use cases for pubmed2ensembl

pubmed2ensembl allows integrated queries over text in PubMed and genomic information in Ensembl *via* human and programmatic web interfaces that take three general forms: (i) text-based (or PMID-based) queries resulting in genomic data, (ii) genomic queries resulting in PMIDs (or links to text), and (iii) integrated queries constrained by both text (or PMIDs) and genomic data. To illustrate the utility of pubmed2ensembl, we provide two brief use cases demonstrating novel features of our system for biologists working in the area of genetics and genomics.

#### Obtaining a list of genes discussing a PubMed search term

A common task in biological data exploration is to generate a list of genes that are associated with a particular biological phenomenon. In principle, this could be achieved using the Gene Ontology (GO) [24], however not all biological phenomena are represented in the GO, nor are all genes fully annotated with relevant GO terms. Likewise, while it is possible to query PubMed and navigate on a paper-by-paper basis to genes in Entrez Gene, it is not possible to generate a list of Entrez genes directly from a free-text PubMed search at the NCBI website. Executing cross-domain queries in pubmed2ensembl is straightforward using our enhanced MartView interface. For example, a developmental biologist can obtain a list of genes with transcriptional enhancers involved in the *D. melanogaster* process of stripe formation during embryonic segmentation as follows:

- choose “Drosophila melanogaster” as species dataset in the pubmed2ensembl BioMart,
- under Attributes, select “Ensembl Gene ID” and “Associated Gene Name” under GENE and de-select “MEDLINE: PubMed ID” under PUBMED2ENSEMBL FEATURES,
- in the NCBI's esearch box at the top of the page, enter “segmentation stripe enhancer”, and select the “search for PubMed IDs” and “filter on Entrez: PMID” drop-down menus,
- click “Search”,
- select the “View” option to 50 rows and select the “Unique results only” check box.

This query will return a list of 23 genes (e.g. *bicoid*, *even-skipped*, *knirps*) that are all known to contain, or regulate, enhancers of segmentation genes that generate stripe patterns in the *D. melanogaster* embryo [25].

#### Obtaining publications for a list of genes or specific genomic interval

Another powerful feature of pubmed2ensembl is that it provides a simple interface to construct complex queries based on lists of genes that result in lists of publications with their corresponding data source for browsing or further data mining. Moreover, because genes have defined genomic locations, lists of genes can be automatically transformed into lists of genomic coordinates in order to obtain publications that pertain to a specific region of the genome. For example, a geneticist performing a deficiency screen in *D. melanogaster* can obtain a list of publications discussing the genes in a molecularly defined genomic interval deleted from DrosDel stock Df(1)ED6991 [26] as follows:

- choose “Drosophila melanogaster” as species dataset in the pubmed2ensembl BioMart,
- under Filters, select the check box for “Chromosome” and select “X” from the drop-down menu under REGION,
- under Filters, select the check box for “Base Pair” and enter “9580686” for Gene Start and “10105557” for Gene End under REGION,
- under Attributes, select the “Ensembl Gene ID” and “Associated Gene Name” check boxes under GENE,
- under Attributes, select the attributes “Entrez: PubMed ID(s) [List]” under PUBMED2ENSEMBL FEATURES,
- click “Results”.

This query will return a list of 42 *D. melanogaster* genes (40 of which are associated with publications) and direct links to PubMed abstracts, which a researcher can quickly browse for relevant published phenotypes that are associated with genes in this genomic interval.

## Discussion

### To what extent are the biomedical literature and genes in Ensembl linked?

As the quantity of information contained in the biomedical literature and biological databases increases, so does the need to assess the status of links between these resources and improve these links where possible [7], [27]. This need is widely appreciated from the standpoint of adding value to curated database records by providing link-outs to relevant literature [7], [12]. However, there is also increasing awareness for the need to provide links in the reverse direction, from publications to databases, as efforts to extract entities and relations from the biomedical literature improve and motivate new approaches to store and search text-mined data [28], [29]. Our view is that the critical link to establish between the literature and bioinformatics databases is at the level of the genes, as most biological databases use genes as their fundamental entity for storage and retrieval. Thus, to assess the state of the integration of the literature with databases, it is important to address the question of how connected genes are to publications, and *vice versa*.

Miller *et al.* [27] have previously addressed the question of how linked GenBank records (which may or may not correspond to genes) are to PubMed entries. These authors found that 42% of GenBank records had a linked publication, but only 71% of these publications were found in PubMed, resulting in only 30% of all GenBank records being associated with a PMID. Assessment of the converse, how many PubMed records have a link to GenBank record was not reported by these authors. Our work provides some preliminary insight into how connected publications in PubMed are to genes in Ensembl in both directions. Overall, we find that the mapping of the biomedical literature to genes in Ensembl is sparse in terms of the total set of possible mappings: only 11.3% of publications with a PubMed ID could be linked to genes in Ensembl and only 13.6% of genes in Ensembl were linked to publications. Assuming these values are reasonable approximations of reality, we interpret the relatively low percentage of publications linked to genes to arise from that fact that a large subset of PubMed is made up of publications on clinical trials, case studies, etc. that do not include information on genes. Conversely, we interpret the relatively low percentage of genes linked to publications to represent the highly biased nature of publication on a subset of well-studied genes. While inclusion of more species beyond those currently in Ensembl is likely to lead to an increase in the proportion of linkable papers, it is not likely to lead to an increase in the proportion of linkable genes. This is because the main focal organisms that are already included in Ensembl have several orders of magnitude more publications on their genes than other species (Figure 3), and thus we may have covered the bulk of the literature on genes, at least for metazoan species.

Whether these estimates are close to the upper limit of the number of possible links between genes and the literature remains an open question. The relatively low degree of overlap between gene-PMID links from different data sources (Table 3) and low recall for most data sources relative to human-curated gold standard (Table 4) suggests that either there are a large number of real gene-PMID links missing from most data sources (false negatives), or that each data source generates a large proportion of false positive links. We favor the interpretation that pubmed2ensembl data sources have many false negative links, since precision for most data sources is high (Table 4). In support of this view, Miller *et al.* [27] show that many real connections to the literature are not found in GenBank records because of inconsistencies in author submission practices and exchange of information between EMBL and GenBank. The possibility that individual data sources are as incomplete as our work and the work of others' suggests further demonstrates the utility and importance of the type of integrative approach to genomic literature mining that pubmed2ensembl offers.

### Which data sources are best for linking genes to the literature?

Determining which data source is the best for researchers to obtain relevant papers on genes of interest can be framed in terms of coverage and quality. As show in Supplementary Files S1, S2, and S3, sequence-based sources (EMBL BLAST, text2genome) permit the broadest coverage of gene-PMID links across species, since mapping of publications to genes does not require curation, nor does it rely on the mapping of gene IDs across databases. Coverage across species of sources that use database cross-references (Entrez Gene, EMBL XREFs) is incomplete because they rely on the availability and consistency of mappings of gene IDs between databases. For example, Ensembl does not provide cross-references in the object_xref table between Entrez Gene ID and Ensembl Gene IDs for 26 of the 50 species of release 56, and four species (cow, dog, chicken and chimpanzee) have Entrez Gene cross-references at the transcript rather than the translation level, as was used in our BioMart construction. Likewise, EMBL only provides cross-references to Ensembl genes for species that are part of the Ensembl gene build, but not for species where gene models are imported from external databases (e.g. fruitfly, worm, yeast). Finally, our data sources that rely on automated gene name recognition are limited by fact that GNAT is capable of finding genes for a set of 18 model organisms [18], but only ten of these are part of Ensembl 56. Moreover, for two of these (cow and chicken), we did not generate Entrez to Ensembl Gene ID mappings because of inconsistencies in the Ensembl object_xref schema noted above. Application of gene name recognition software, such as the recently released GeneTUKit system [30], that are not restricted to particular species would improve species coverage for our text mining data sources. However, mapping issues between Entrez and Ensembl gene IDs remain a limiting factor for the coverage and automated linking of text mining data sources across species.

The quality of the data in pubmed2ensembl can be estimated from our analysis of gene-PMID links versus human curated data from the BioCreAtIvE challenges for four model organisms (Table 4). The generally high precision and low recall values in this analysis indicate that the gene-PMID links provided in pubmed2ensembl are indeed accurate but that many possible gene-PMID links are missing from our data sources. The exact values of recall and precision for some data sources should be interpreted with caution, since the document type (e.g. abstracts versus full text) and availability (OA versus all articles) can differ between pubmed2ensembl data sources and the BioCreAtIvE evalutation set (abstracts only). For example, Entrez Gene links are based on curating full-text articles and not only abstracts, which may explain the relatively high number of putative FPs in this dataset. For the pubmed2ensembl data source most closely matched in structure to the evaluation set (MEDLINE), precision is very high (>85%) but recall varies according to the species (from 3% in fruitflies to 63% in humans). This observation raises the general point that the quality of gene-PMID links varies across species. Differences in quality across species arise indirectly from differences in the coverage of data sources across species (see above), but also from species-specific differences in the ability of some data sources to generate gene-PMID links. For example, fruitfly gene names are harder to detect using text-mining approaches because of their similarity to English words [23], which likely explains the low recall for the MEDLINE data source in this species.

### Relationship of pubmed2ensembl to other resources

Finally, it is useful to place pubmed2ensembl into the ecology of related resources that attempt to provide integration between the biomedical literature and genomic data to help users identify relevant systems that best suit their particular research objectives. For relevance we restrict our focus here to active systems available on the web, and exclude several systems that are no longer maintained or that require command-line access [31], [32], [33], [34]. Although not detailed fully in the following discussion, it is important to re-emphasize here that pubmed2ensembl inherits many powerful features of the generic BioMart framework [14] that separate it from similar resources that lie at the interface of database and literature integration (see Materials and Methods).

The first set of systems that are related to pubmed2ensembl are those that provide an integrated search interface over the literature and genomic data, such as Entrez (http://www.ncbi.nlm.nih.gov/books/NBK3837/) or the Sequence Retrieval System (SRS) [35]. Both Entrez and SRS provide the ability to perform text searches across both literature and genomic databases, and the ability to search on either literature or genomic databases and navigate from one to the other *via* internal database cross-references. Like pubmed2ensembl, it is possible to query Entrez or SRS with a list of PMIDs or Entrez Gene IDs, but only formatted using a Boolean ‘or’ search rather than a true ID list. However, neither Entrez nor SRS provides the ability to search on one database (e.g. PubMed) and directly return data from another database (e.g. Entrez Gene), even though cross-references between different databases are stored internally. Furthermore, identifiers in the Entrez system are not unique across databases, so it is not possible to query multiple databases simultaneously in Entrez with a list of numeric PMIDs or Entrez Gene IDs and retrieve unambiguous search results. Finally, and most critically, it is not possible to combine either Entrez or SRS text searches with genomic constraints (such as chromosomal locations), as is trivial to do in the pubmed2ensembl BioMart.

Other systems, such as the Hyperlink Management System (HMS) [36] or bioDBnet [37], provide a fixed set of ID mappings between PubMed and Ensembl genes. Users can provide the HMS with a list of Ensembl (or other) gene IDs and retrieve PMIDs, and vice versa. However, HMS only supports links for three species (human, mouse, sea squirt) and cannot perform text based queries. bioDBnet is not restricted in terms of the species it provides mapping for, and it allows a text search on Entrez Gene database records, but not on the associated PubMed abstracts as is possible in pubmed2ensembl. Critically, the provenance of these mapping is not entirely transparent in either system. Furthermore, neither HMS nor bioDBnet utilize the wealth of gene-PMID links provided by text mining, and neither have the capability to construct integrated queries with text and genomic data as is possible in pubmed2ensembl.

Text mining has been used to map gene IDs to publications in several data-mining systems, such as iHop, GoGene, QuExT or MarkerInfoFinder. The iHop system [38] allows users to search for single gene names or IDs and returns excerpts of MEDLINE abstracts containing that gene. In contrast to pubmed2ensembl, iHop does not support free text searches or lists of genes/PMIDs as input, nor can gene-PMID links be integrated with other genomic resources or searched with genomic constraints within iHop directly. Furthermore, while providing data for individual genes automatically by web services [39], gene-PMID links in iHop are not publicly available for bulk download. The GoGene system [40] allows users to input a list of Entrez Gene IDs, protein sequences, or a free text query to PubMed, and based on the underlying list of gene-PMID associations, returns ontology terms found in the text organized using the GoPubMed interface [41]. As a byproduct, GoGene generates a list of gene-PMID links for a given query, however queries cannot be joined with genomic constraints or produce related genomic information. QuExT [42] takes gene lists input by a user, expands gene names to include synonyms, and uses the expanded gene list to rank publications for relevance based on gene name recognition in PubMed. QuExT cannot use free text or genomic constraints as input to searches, and only provides output *via* a non-programmatically accessible web-interface. Finally, MarkerInfoFinder [43] uses text-mining to link gene names and other genetic markers (e.g. SNPs, cytological locations) to MEDLINE records. Using this data, MarkerInfoFinder provides a web interface to search lists of gene names or a single coordinate range in the human genome for corresponding MEDLINE records, but does not provide the ability to conduct queries with text or other genomic constraints.

The last set of resources that provide related functionality to pubmed2ensembl are those that attempt to integrate results of text mining on the biomedical literature with genome browsers, including text2genome, FABLE/LitTrack and PosMed. As noted above, text2genome [21] extracts sequences from full text articles and provides links between genes and genomic locations. text2genome provides a simple a text search interface and limited API to query for gene names, keywords or PMIDs/PMCIDs, but does not directly provide the ability to construct complex queries over text and genomic data. FABLE (http://fable.chop.edu) uses text mining to normalize mentions of genes in PubMed abstracts to Entrez gene IDs and provides a free text search that returns a gene list with links to articles. The FABLE system also serves a customized UCSC genome browser (FABLE's LitTrack) with a track displaying the papers linked to a gene, similar to our pubmed2ensembl DAS source. However, users cannot construct complex queries over text and genomic data in FABLE or LitTrack. Finally, the Positional MEDLINE (PosMed) system [44], [45] is a database and query system based on textual and genomic data used to prioritize candidate genes in positional cloning experiments. PosMed uses a simple text mining strategy base on rule-based searches to construct a database of gene-PMID links that are joined with a database of PMID-keyword links to find associations of keyword with genes. Users can use text queries in conjunction with genomic constraints to generate a ranked list of candidate genes that can be browsed or download in fixed formats. In contrast to pubmed2ensembl, PosMed cannot use PMID lists as input (or return them as output), and there is no ability for users to construct queries or return data in a customized manner as is possible in a BioMart.

### Conclusions and future directions

Here we have shown that information from the biomedical literature can be effectively linked to genes in Ensembl and warehoused in a customized BioMart for integrated queries over text and genomic data. We have also shown that the open source code base of the BioMart system flexibly permits customizations that allow the integration of text and genomic data, and that the BioMart construction process can be carried out fully automatically. We have characterized six different sources of gene-PMID links for integrating published information on genes into an extended Ensembl BioMart, and given preliminary insights into the quantity and quality of links that can be made between genes and literature. We have identified several related resources that attempt to bridge the gap between the rapidly growing amount of information on publications and genes, but conclude that the native functionality and common interface of the BioMart framework coupled with the innovations presented here present a powerful and unique resource for mining the biological literature on genes.

We imagine extending pubmed2ensembl in several directions in the future. First, using the BioMart 0.7 framework we could include additional data sources, such as those based on different automated gene name normalization tools or those based on other databases of curated gene-PMID links. We could also attempt to find new sources of gene-PMID links such as applying gene name recognition on database records or by attempting to mine database identifiers, genetic markers or mutations from text. With current or enhanced data sources, another direction to pursue would be to incorporate publication information directly into the main Ensembl BioMart at EBI to allow the full utility of the pubmed2ensembl approach to reach the widest possible audience. Finally, features of the pubmed2ensembl 0.7 BioMart could be implemented in more modern data mining systems such as the InterMine framework [46] or BioMart 0.8, which has the benefits of easier configuration, a new Java API, and a semantic web interface that enhances the ability of researchers to access, integrate and utilize published information on genes and genomes.

## Supporting Information

File S1 Supporting information.
(TXT)

File S2 Supporting information.
(TXT)

File S3 Supporting information.
(TXT)

File S4 Supporting information.
(TXT)

File S5 Supporting information.
(TXT)

File S6 Supporting information.
(TXT)

File S7 Supporting information.
(TXT)
